# Mixed-Phenotype Acute Leukemia: Clinical Diagnosis and Therapeutic Strategies

**DOI:** 10.3390/biomedicines10081974

**Published:** 2022-08-15

**Authors:** Binsah S. George, Binoy Yohannan, Anneliese Gonzalez, Adan Rios

**Affiliations:** Department of Hematology/Oncology, McGovern Medical School, The University of Texas Health Science Center at Houston, 6431 Fannin Street, MSB 5.216, Houston, TX 77030, USA

**Keywords:** acute lymphoblastic leukemia-type therapy, allogeneic transplantation, genetic alterations, mixed-phenotype acute leukemia, targeted therapy, ambiguous leukemia

## Abstract

Mixed-phenotype acute leukemia (MPAL) comprises a heterogenous group of leukemias that are genetically, immunophenotypically, and clinically, diverse. Given the rarity of the disease, the diagnosis and treatment of MPAL is extremely challenging. Recent collaborative efforts have made significant progress in understanding the complex genomic landscape of MPAL. Some retrospective studies support starting ALL-type induction followed by an allogeneic stem cell transplant(allo-sct) in the first complete remission; however, due to the inherent bias of retrospective data and small case series, a prospective validation of AML- and ALL-based regimen, and the incorporation of targeted therapies based on genetics and immunophenotype are warranted. The prognosis of adults and children with MPAL varies; this justifies modulating the intensity of therapy, including the use of allo-sct as a consolidation strategy.

## 1. Introduction

Acute leukemia is often myeloid or lymphoid in origin. Occasionally, leukemia blasts can express both acute lymphoblastic leukemia (ALL) and acute myeloid leukemia (AML) markers, characterized as biphenotypic or bilineal blasts. The World Health Organization (WHO-2008) classification of hematopoietic and lymphoid tumors defined this type of leukemia as mixed-phenotype acute leukemia (MPAL), and it represents 1–3% of acute adult leukemias. Nearly 40 years ago, this entity was described as “leukemia of ambiguous origin”; however, over the years, several nomenclatures and algorithms have been introduced to better define leukemia [1,2,3].

The European Group of Immunological Characterization of Leukemia (EGIL) classification was proposed in late 1990s based on a well-validated scoring system that requires two or more points in two separate lineages for diagnosing MPAL (Table 1) [4,5,6].

Immunophenotype criteria for lineage classifications in MPAL according to EGIL and WHO classification systems.

In 2008, the WHO classification acknowledged lineage-specific antibodies and genetic markers; this was last updated in 2016 and uses a total of 10 antibodies for diagnosis (Table 2) [7,8].

Other WHO-defined leukemias, such as AML with myelodysplasia-related changes (AML-MRC), therapy-related AML, blast-phase chronic myeloid leukemia, or AML with balanced translocations, such as core-binding leukemia [t(8;21), inv(16)] and PML-RARA t(15;17), are excluded from MPAL; this is because it has been recognized that these types of leukemia can present with an MPAL immunophenotype. The presence of three or more aberrations is considered an indication for a complex karyotype and is the most common genomic anomaly in MPAL. However, AML-MRC is often characterized by a complex karyotype including monosomy 7 and 5; these mutations are recurrently added to the MPAL series. It is controversial whether such patients should be eliminated from the MPAL category, as they fulfill criteria for an alternative WHO AML diagnosis or be retained in the classification if leukemia blasts otherwise meet the criteria for MPAL [9,10,11]. In a study of MPAL in children with a complex karyotype, the majority of patients responded well to ALL-directed therapy [12,13].

The current 2016 updated WHO classification for MPAL is subdivided into two genomic categories: BCR-ABL fusion–positive and KMT2A-rearranged (KMT2Ar, formerly MLL) cases. The remaining types of MPAL are reported based on immunophenotype B/myeloid (59%), T/myeloid (35%), or rarely, T/B (4%), T/B/myeloid (2%), or phenotype (Table 3) [7,8,14].

According to the SEER database, MPAL carries the worst outcomes among adult leukemias [15]. Due to the rarity of MPAL, treatment guidelines are not clearly established. The current treatment recommendation is an ALL-like induction regimen followed by allogeneic stem cell transplant (allo-sct) in the first complete remission (CR1); however, due to the inherent bias of retrospective data and small case series, a prospective validation of AML- and ALL-based regimens is warranted. Therapies beyond induction are not well established. MPAL with KMT2A can switch between lymphoid and myeloid lineages, and such lineage pliability is one of the important reasons for the aggressive nature of MPAL and subsequent poor outcomes [16].

This review will focus on innovative methods to improve current treatments using the understanding from recent developments in genetic analysis, immunophenotypic, and clinical insights. The exploration of more powerful tools as in artificial intelligence, machine learning, and improved flow cytometry (FC) generates avenues to improve survival outcomes in MPAL [17].

## 2. Discussion

### 2.1. Immunophenotypic and Genetic Classification

Generally, immunophenotypic analysis, including FC, cytochemistry, and immunohistochemistry (IHC), is required for a precise diagnosis of MPAL. Morphologically, MPAL blasts have a hand-mirror morphology, small to intermediate size, and variable cytoplasm. Occasionally, the 2 blasts could have a distinctive phenotype, morphology, and size [18].

The WHO does not mandate a certain percentage of lineage-specific antigens, unlike in EGIL, and depends on the intensity of antigen expression. The WHO stipulates a myeloid lineage in MPAL with the detection of MPO by cytochemistry, FC, monocytic differentiation, or IHC, and when blasts meet the criteria for B or T lineage [8]. In addition, it is not uncommon to have MPO mRNA positivity in otherwise typical ALL [19,20,21]. The reverse transcription-polymerase chain reaction is the most sensitive test to detect MPO, and this is followed by immunohistochemistry; however, FC is less sensitive to IHC and enzyme cytochemistry is the least sensitive. Thus, depending on the methodology used to detect MPO, the diagnosis of MPAL can vary. To address this issue, efforts are ongoing to standardize MPO positivity. Many studies have agreed on a cutoff of MPO positivity by FC varying from 5.4% to 20%, and by an enzyme cytochemistry of 3% (not formally established) [13,22,23].

Around 42% of B-ALL cases that express MPO show evidence of t(9;22) translocation, are likely to express CD13 or CD15, and are associated with a chronic myeloid leukemia blast phase (CML-blast) rather than de novo ALL [20]. Some Burkitt-like entities have strong MPO staining by cytochemistry or FC; however, they lack CD33, CD13, and CD117 expression [24]. Cases of pediatric B/myeloid MPAL with expression of MPO and CD15, CD33, or CD13 with B-ALL related cytogenetics as ETV6-RUNX1, and trisomy 10 and 4, seem to do well with an ALL-type regimen [25].

Cytoplasmic cluster of differentiation (cCD3) is essential to assign a T-cell lineage in MPAL. CD2, CD7, and CD56 are other T-cell markers that are found in many AMLs. The cCD3 fluorescence determined by fluorochrome should be comparable to that of normal T cells [8,26]. Natural killer (NK) cell lymphoblastic leukemias can express cCD23. Terminal deoxynucleotidyl transferase (TdT) is a ubiquitous marker; however, it is most commonly expressed in ALL [27].

In the case of B lineage disease, a strong CD19 expression with another marker or weak CD19 expression with 2 other markers (CD 10, CD79a, or cytoplasmic CD22) is indicative; thus, this makes CD19 a mandatory marker, but not adequate for diagnosis. Of note, CD19 is expressed in 7% of all non-acute promyelocytic leukemias, including AML with t(8;21), RUNX1 mutation, and NPM1 mutation. Other B cell markers, such as CD10, CD22, CD79a, and PAX5, are expressed in AML and acknowledged in the WHO classification [28,29]. Although CD22 is expressed in other cells such as basophils, it is considered a specific marker for MPAL by the WHO. Expression of CD19 is variable in de novo versus relapsed leukemia, and is frequently negative after blinatumomab and chimeric antigen receptor (CAR) T-cell therapy [30,31].

The understanding of lineage-specific expression in the context of varied genetic and cytogenetic abnormality continues to remain unclear. A clear distinction could improve therapeutic management and outcomes.

In addition to immunophenotype, molecular analyses have become more common in the characterization of MPAL. Genetic alterations seen in AML and ALL are frequently documented in MPAL; thus, they describe its genomic integration and heterogeneity. A median of 2 (range, 0–7) mutations are seen in MPAL, which is similar to the number of mutations detected in AML or T-ALL; however, MPAL has more mutations than B-ALL. The mutational profile of B/myeloid MPAL is different from that of T/myeloid MPAL [32]. The T/myeloid type bears more frequent mutations and a less complex karyotype than B/myeloid MPAL [33].

Mutations in T/myeloid MPAL frequently involve the IDH2, WT1, CEBPA DNMT3A, EZH2, PHF6, FLT3, KRAS, NRAS, CDKN2A/B, ETV6, NOTCH1, IL7R, FBXW7, and JAK/STAT signaling proteins. Early T precursor ALL (ETP-ALL) is a subgroup of T-ALL in which NOTCH1 mutations are frequently reported. ETP-ALL has poor outcomes and is considered a form in between T/myeloid MPAL and T-ALL. A notch-1 mutation is reported in 10–15% of T/myeloid MPAL, which is slightly less than in T-ALL and ETP-ALL [32,34,35].

B/myeloid MPAL has mutations in RUNX1, ASXL1, EZH2, and TET2, and deletion in IKZF1 (Ikaros) [3]. ZNF384 rearrangements (with EP300, TCF3, TAF15, and CREBBP) are reported in nearly 40% of pediatric B/myeloid MPALs; however, they are not reported in adult MPAL. NUP98 and PICALM genes are the other gene rearrangements observed in MPAL [32,35]. A PICALM mutation is reported in AML and T-ALL, including MPAL, and usually observed in young men with extramedullary disease [36].

### 2.2. Therapeutic Approach

The rarity of MPAL with the capability to switch lineages under pressure (treatment) defines the challenges in treating MPAL. The debate on the most appropriate induction regimen remains unresolved over the controversy whether initial therapy should be based on immunophenotype, cytogenetics, or molecular biology. MPAL has a poor prognosis and worse outcomes than standard-risk AML or ALL [15]; multivariate Cox regression analysis of SEER registry data from 316 patients with MPAL reported an increase in death by 59% compared with ALL and by 26% with AML [12,15]. Clinical outcomes of children with MPAL tend to be better when compared to adults with MPAL, with a higher overall survival (OS) and fewer relapses; however, outcomes for acute leukemia in general are better in children than in adults [15,37,38]. The series of questions faced by any provider having to treat MPAL are: the best induction regimen to attain remission; early signs of treatment failure; possible salvage regimens who fail induction/consolidation; and the role of allo-sct.

However, despite the setbacks, there are multiple ongoing efforts to improve outcomes in MPAL. A growing preponderance of data suggests that an ALL-based induction regimen followed by allo-sct in CR1 stands the greatest chance of benefit. However, if the patient fails induction, treatment can be switched to an AML-like regimen followed by allo-sct in CR1 [25]. Recently, a few case series/reports of deep remission have reported on this post-induction treatment with an AML-like regimen [39,40,41].

In a series of retrospective studies, the superiority of ALL-like regimens in pediatric and adult patients has been reported; however, these studies are limited by selection bias, retrospective analysis, and small patient numbers [42,43,44].

In one large case series of 233 pediatric MPAL cases reported by the international Berlin-Frankfurt-Munster Study of Leukemias of Ambiguous Lineage (iBFM-AMBI2012), the OS was significantly higher in patients receiving an ALL-like regimen than in those who received AML-like therapy or a combination/hybrid regimen. The 5-year EFS was 80% ± 4% for patients who received ALL-type therapy compared to 36% ± 7.2% for AML-type or combined-type treatments (50% ± 12%). For patients who were positive for CD19, an ALL-like regimen did especially well (5-year EFS, 83% ± 5.3%). Similarly, patients positive for other lymphoid markers, such as CD3 and CD7, or for at least two of the following—CD22, CD10, and CD79, but who were negative for CD19, did well on an the ALL-like regimen. In the study, the only subset of MPAL patients (*n* = 10) that benefited from an AML-like regimen were CD19 negative with no other lymphoid features [45].

In a report of 100 patients, two-thirds of whom were >15 years old, an ALL-like regimen resulted in an improved survival over an AML-like regimen or a hybrid ALL/AML combined regimen [37].

A recent meta-analysis of 1300 patients, inclusive of international small series and case reports of patients diagnosed with MPAL(WHO) or biphenotypic leukemia (EGIL), reported that patients receiving an induction regimen with ALL-like therapy were significantly more likely to achieve complete remission (CR) and twice less likely to die than patients receiving an AML-like regimen (MPAL(WHO-2008) odds ratio [OR] = 0.33; 95% confidence interval, 0.18–0.58). However, in a multivariate analysis of detailed clinical data, including treatment type, MPAL subgroup, and patient age, there was no OS difference between ALL and AML induction regimens (3-year OS, 48% ± 6.9% vs. 47% ± 5.0%, respectively). MPAL patients who received hybrid regimens (combined ALL and AML therapy) had significantly worse survival (3-year OS, 23% ± 8.6%, *p* = 0.001), perhaps due to increased toxicity [38].

Similar results were reported by a Children’s Oncology Group (COG) study that retrospectively reviewed pediatric MPAL patients who strictly passed through the central review of WHO-defined MPAL in which the 5-year OS were not different between ALL and AML type regimens (78% ± 9% vs. 69% ± 14%, *p* = 0.548) [46].

Recent case reports have noted efficacy and successful outcomes with a FLAG (fludarabine, cytarabine, and granulocyte colony-stimulating factor) and venetoclax-based induction regimen. In older patients who were ineligible for intensive chemotherapy or allo-sct, the use of a hypomethylating agent (HMA) with venetoclax induced CR with tolerable toxicities [39,40,41]. A hybrid regimen of FLAG-IDA (idarubicin)-vincristine-prednisone in 8 MPAL patients rendered a 1-year OS of 85.7%, and all patients achieved CR/CRi (complete remission with incomplete hematological recovery) [47].

Recently, the integrated genomic analysis of 31 adult MPAL patients was compared to that of AML and ALL patients, and methylome expression analysis segregated T/myeloid MPAL and B/myeloid MPAL to T-ALL and AML-like leukemia, respectively. CR was more likely if therapy matching the methylome was administered, with remission rates of 72% for matched and 22% for unmatched therapy (*n* = 27, *p* = 0.037). However, the composite CR rates (*p* = 0.58) and OS (*p* = 0.25) rates were similar between the matched and unmatched group [32].

In summary, given the limitations of available retrospective data, the general consensus is to use an ALL-like induction therapy for pediatric and adult MPAL. Validation from prospective studies is required to understand the subset of MPAL patients that best responds to an AML- versus an ALL-like approach.

Measurable residual disease (or minimal [MRD]) assessment is one of the most common prognostic indicators for therapeutic decision-making in ALL. In the iBFM AMBI2012 trial of pediatric MPAL, early eradication of MPAL clones at the end of induction (EOI) had improved EFS. MPAL patients (CD19-positive) with a positive MRD (>0.01%) by the end of 12 weeks had a worse EFS of 50% ± 19% compared to 83% ± 5.3% for the entire CD19-positive cohort treated with an ALL-like regimen [45]. In data reported by the COG of children with B/myeloid MPAL (*n* = 53) treated with an ALL-like regimen, a flow-based MRD of <0.01% at the EOI was the most important predictor of EFS in multivariate analysis (hazard ratio 5.0, 95% CI 1.7–15.3, *p* = 0.0036) [48]. Moreover, patients who had early MRD negativity at the EOI had a better OS when compared with those who had MRD clearance at the end of consolidation [49].

### 2.3. Targeted Therapy

In patients with BCR-ABL fusion positive MPAL, the addition of a tyrosine kinase inhibitor (TKI) to the chemotherapy backbone is highly recommended. Patients with Philadelphia positive (Ph+) MPAL who received TKIs had a significant reduction in the risk of death in comparison to Ph (-) MPAL patients (hazard ratio [HR] = 0.28, *p* = 0.002) [50,51]. However, the KMT2Ar subset of patients showed conflicting results: some reports show no difference in survival [38,52], whereas recent data show poorer survival with a tenfold increase in death compared Ph+ MPAL (HR = 10.2, *p* < 0.001) [35,51]. Incorporating investigational therapies that address the biology of KMT2A, such as DOT1L, bromodomain, or a menin inhibitor, could improve survival in these patients [53].

Targeted approaches can be used in high-risk disease, especially in patients with MRD and in the relapsed refractory setting. Targetable pathways of frequently activated mutations, such as FLT3 through ITD and TKD, would benefit from a FLT3 inhibitor [54,55]. A recent report suggests that the FLT3 mutations are more commonly seen in a T/myeloid phenotype with 7 of 15 patients harboring FLT3 mutations; the majority were CD117+, and this conclusion was further validated in the larger pediatric cohort from St. Jude’s. B/myeloid MPAL patients with ZNF384r and MLLr MPAL can exhibit FLT3-mediated signaling even when a somatic FLT3 mutation is absent [35,56]. IDH1 and IDH2, which are epigenetic regulators, are useful targets in this case [57,58]. Agents that target CD19, such as blinatumomab (a bispecific CD3/CD19 monoclonal antibody) or inotuzumab ozogamicin for CD22, can also be used [59,60,61]. CAR-T therapy engineered to target CD19-positive blasts is an emerging option with some success in case reports [62,63]. Venetoclax, which targets BCL-2, combined with various chemotherapies in MPAL, has shown encouraging results in recent case series [39,64,65]. Magrolimab, a macrophage checkpoint inhibitor targeting CD 47, in combination with azacytidine, has shown promising activity in high-risk AML. In addition, T-cell immunoglobulin and mucin domain-containing protein 3 (TIM3) is expressed in leukemic stem cells and offers an exciting new target in AML. Investigational immune myeloid inhibitors such as Sabatolimab have shown promising antileukemic activity in early phase trials. Several monoclonal antibodies targeting CD 70, CD 45, CD 123, and CD33 are currently being investigated in myeloid neoplasms. The novel immunotherapies could potentially change the therapeutic landscape of acute leukemias in the future [66].

### 2.4. Salvage Therapy

Given the lack of prospective trials and rarity of the immunophenotypically complex MPAL, diagnosing treatment failure may be challenging from the emergence of subclones, lineage switch, clonal shift, or small immunophenotypic residual population. The most commonly used approach is changing therapy to an AML-like regimen if induction therapy used an ALL-like regimen, and vice versa. Additive toxicity is a concern, however. In a recent pediatric case series of MPAL with MRD, there were no adverse outcomes; nonetheless, this may be rather debilitating in older patients [52]. A few case reports and series have described using targeted therapies, including nelarabine [66,67], blinatumomab [67,68], inotuzumab ozogamicin, and CAR-T infusion [68,69], which would be better tolerated in elderly patients. The challenge in using CAR-T therapy in MPAL is a concern for lineage switch and immunophenotypic plasticity. AML-based regimens, such as FLAG-IDA with venetoclax, or a hypomethylating agent (HMA) and venetoclax, hybrid regimens such as FLAG-IDA-vincristine, and prednisone with or without rituximab, are other options with the goal of allo-sct during CR1 or CR2 in eligible patients [39,40,41,47].

### 2.5. Allogeneic Stem Cell Transplantation

Allo-sct is usually reserved for acute leukemia patients with high-risk cytogenetics; MPAL has historically been regarded to have high disease resistance and inferior survival. In a retrospective review of 500 adult patients with MPAL, reported in the European Society of Blood and Marrow Transplant Research registry, the 3-year OS for patients that underwent allo-sct was 56%. When total body irradiation (TBI) was included in the conditioning regimen, survival was improved compared with other myeloablative regimens [69,70]. According to the reports from the Center for International Blood and Marrow of 95 patients (78 and 17 patients in CR1 and CR2, respectively) with MPAL who underwent allo-sct, the 3-year OS, leukemia-free survival, relapse incidence and non-relapse mortality were 67%, 56%, 29%, and 15%, respectively. A matched analysis comparing MPAL cases to AML or ALL cases showed similar outcomes. The OS of patients who underwent allo-sct in CR1 versus CR2 were similar [70,71]. In adult MPAL, the data suggest that allo-sct during CR1 can result in outcomes similar to those of ALL and AML patients. Conversely, in pediatric MPAL, an early response to ALL-like therapy contraindicates allo-sct; it is reserved only for relapsed refractory disease or patients with MRD ≥ 5% after initial induction [45]. On the basis of current data, adult patients with MPAL are recommended to proceed with allo-sct during CR1 if a donor is available. As in ALL, the role of MRD-guided allo-sct is not clear, and more data from prospective clinical trials is needed.

### 2.6. MRD Assessment

The assessment of MRD is challenging in MPAL due to its variable immunophenotypes and the emergence of subclones during treatment. In the largest series of 233 patients in the iBFM-AMBI2012 retrospective analysis, it was noted that patients with an MRD ≥ 5% at the EOI therapy had a 5-year EFS of <50%. Current guidelines on the treatment of MPAL mainly suggest an ALL-focused regimen unless the patient does not express CD19 and does not have expression of B-cell markers according to immunophenotyping. Hence, the COG has recommended using an MRD cutoff of <5% post-induction and <0.01% post-consolidation to carry on with any planned ALL-based regimen. Patients who do not reach these target goals would benefit from a switch in therapy to an alternative AML-type regimen, or early intensification followed by a suitable donor search for allo-sct consolidation [45].

## 3. Conclusions

Redefining the classification and discovering the genomic underpinnings of MPAL are some of the significant advancements that have been made in the past decade. Contextualizing the emerging understanding of the biology of MPAL to allow homogeneity in treatment strategies would enable the risk stratification of patients and treatment approaches between MPAL subsets. This would present the opportunity to compare outcomes between ALL- and AML-based regimens and identify patient subsets that are likely to benefit from either therapy. Given the modest success with targeted molecular and immunotherapies in the salvage setting, the next step in advancing the field should include incorporating novel therapies in de novo MPAL.

## Figures and Tables

**Table 1:** European Group of Immunological Classification of Leukemia scoring system.

	2 Points	1 Point	0.5 Points
Myeloid Lineage	Myeloperoxidase Lysozyme	CD117 CD33 CD13 CD65a	CD14 CD15 CD64
B-lymphoid lineage	CD79a cyt IgM cyt CD22	CD19 CD20 CD10	TdT CD24
T-lymphoid lineage	CD3 (cytoplasmic or surface) TCR alpha/beta TCR gamma/delta	CD2 CD5 CD8 CD10	TdT CD1a CD7

**Table 2:** Revised 4th edition World Health Organization (WHO) classification of acute leukemia of ambiguous lineage (ALAL).

Myeloid Lineage	Myeloperoxidase (MPO) (Flow Cytometry, Immunohistochemistry or Cytochemistry) OR Monocytic Differentiation (at Least 2 of the Following: Nonspecific Esterase Cytochemistry, CD11c, CD14, CD64, or Lysozyme)
B-lineage	Strong CD19 and ≥1 strongly expressed marker: CD79a, cytoplasmic CD22, or CD10 OR Weak CD19 and ≥2 strongly expressed markers CD79a, cCD22, or CD10.
T-lineage	Strong cytoplasmic CD3 (strong is equal to or brighter than the normal B or T cells in the sample), CD2, CD5, CD7, and CD8 are commonly expressed in B-ALL and even AML; hence not assigned to T lineage OR Surface CD3 expression.

**Table 3:** Revised 4th edition World Health Organization (WHO) classification of mixed-phenotype acute leukemia (MPAL).

MPAL
MPAL, B/myeloid, NOS
MPAL, T/myeloid, NOS
MPAL, NOS, rare types (T/B/myeloid)
MPAL with gene arrangements
MPAL with t(9;22) (q34.1;q11.2); BCR-ABL1)
MPAL with t(v;11q23.3); KMT2A-rearranged

## Data Availability

Data sharing not applicable.
